# Comparison of antero-posterior view epidurogram for interlaminar epidural steroid injection with contralateral oblique and lateral fluoroscopic views

**DOI:** 10.1016/j.inpm.2022.100160

**Published:** 2022-12-16

**Authors:** Afrin Sagir, Thomas T. Simopoulos, Jatinder S. Gill

**Affiliations:** a Department of Anesthesiology, Critical Care and Pain Medicine, Beth Israel Deaconess Medical Center, Harvard Medical School, 330 Brookline Ave, Boston, MA, 02215, USA; b Department of Anesthesiology and Critical Care, Hospital of the University of Pennsylvania, Perelman School of Medicine, 3400 Spruce Street, Philadelphia, PA, 19104, USA

**Keywords:** Epidural steroid injection, Antero-posterior view, Contralateral oblique view, Epidurogram, Ventral interlaminar line, Spinolaminar line

Dear Editor,

Epidural steroid injections are commonly performed to treat radicular pain [1]. The success of this procedure depends on accurate placement of the steroid within the epidural space, in close proximity to the affected nerve root. The procedure has inherent risks of accidental needle tip advancement to the posterior epidural space, which could result in intrathecal, intradural or intracord injection with devastating consequences, or depositing the medication outside the epidural space in case of false loss of resistance superficial to the epidural space [[2], [3], [4]]. Fluoroscopy is therefore routinely used to ascertain that the medication is correctly placed within the epidural space. Radiographic contrast agent is injected to confirm the needle placement within the epidural space. In this regard it is not clearly known whether a standalone anteroposterior (AP) view can distinguish non-epidural superficial spread of contrast in case of false loss of resistance from the true epidural spread. Contralateral oblique (CLO) view and lateral view by virtue of defining the depth of the needle insertion and contrast spread beyond the ventral interlaminar line and spinolaminar line respectively are more accurately able to distinguish superficial non-epidural spread from epidural spread [4,5]. The multi-society group for safe use of epidural steroid injections recommends obtaining AP and contralateral oblique or lateral view during epidural access [6]. There is evidence to suggest a high incidence of false loss of resistance during epidural access [7]. However, there is no evidence to suggest that this false loss of resistance will be detected by an examination of the AP epidurogram. Indeed, it has been contended that lateral view and/or contralateral oblique view are not routinely needed to confirm epidural placement and the recommendations of the multi-society group are not evidence based [8]. In this article, we describe a case of superficial non-epidural spread that appears like epidural spread in the AP view. We discuss the diagnostic utility of the AP view in comparison to the CLO and lateral views for confirming epidural spread of contrast.

43-years-old man was referred to our pain clinic by his neurosurgeon for an epidural steroid injection prior to considering posterior lumbar interbody fusion surgery at L4-L5 level for disc herniation. The patient presented with cramping pain in his left lower extremity, which was refractory to medications including gabapentin and methocarbamol as well as to physical therapy. Physical examination was remarkable for positive straight leg raise test on the left side. Magnetic resonance Imaging (MRI) of lumbar spine was remarkable for a disc herniation at L4-L5 level with minimal protrusion contacting the left L5 nerve root, and resolution of previously visualized compression of thecal sac and spinal stenosis which was evident in the prior MRI that was obtained three years ago. We decided to perform interlaminar epidural steroid injection at the L4-L5 level with a left sided parasagittal approach. The patient was placed in the prone position, skin was prepped with chlorhexidine and alcohol prep, and draped in a sterile fashion. L4-L5 interlaminar space was visualized in AP view and the site for needle insertion was marked after confirming that this site did not have prior surgery. After anesthetizing the skin with 1 ​ml of 1% lidocaine using a 25G 1.5 inch needle, a 20G 3.5 inch Tuohy needle was guided close to the ventral interlaminar line (VILL) using CLO view. Then loss of resistance (LOR) technique was used to further advance the needle tip, and contrast was injected after obtaining loss of resistance in CLO view; this revealed spread posterior to the VILL which was clearly consistent with non-epidural spread. In the lateral view there was spread along the facet lucency, but a slight posterior extension of the contrast spread pattern pointed to its non-epidural nature. In the CLO view, the contrast spread was posterior to the VILL. However, the AP view had an epidural-like contrast spread pattern, these patterns are shown in Fig. 1. The needle tip was further advanced, resistance was perceived prior to obtaining true LOR, at which time the CLO view showed spread anterior to the VILL and the lateral view showed epidural pattern anterior to spinolaminar line without posterior extension, the AP view continued to have epidural-like spread (Fig. 2). 4 ​ml of the medication (2 ​ml of 40 ​mg/ml methylprednisolone ​+ ​1 ​ml of 1% lidocaine ​+ ​1 ​ml of preservative free normal saline) was then injected into the epidural space and the needle was then withdrawn. The patient had an uneventful recovery after the procedure.

**Fig. 1:**
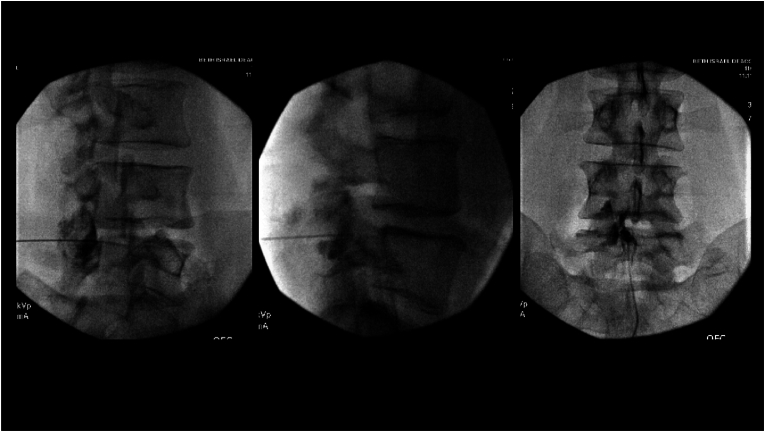
Non-epidural contrast spread pattern after false loss of resistance in the CLO view, lateral view, and AP view after initial loss of resistance. CLO view reveals spread posterior to ventral interlaminar line, lateral view shows epidural like spread however there is posterior extension of contrast pointing to non-epidural nature, however the AP view shows contrast spread has an epidural-like pattern.

**Fig. 2:**
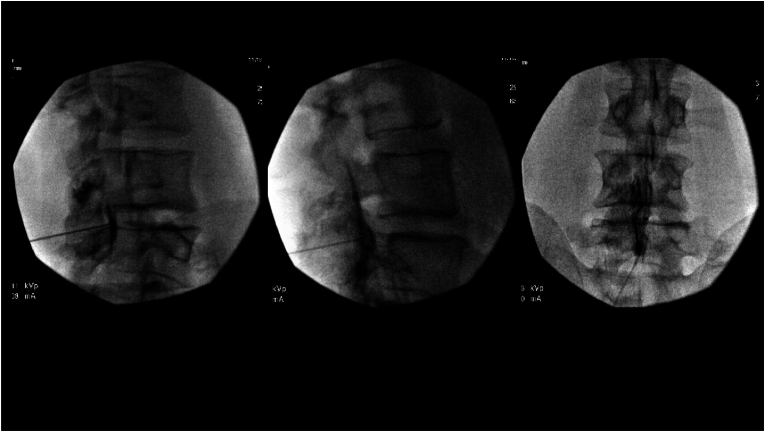
Epidural contrast spread pattern in the CLO view, lateral view, and AP view after advancing needle and subsequent loss of resistance. CLO view shows spread anterior to the VILL, lateral view shows epidural spread pattern without extravasation, while the AP view continues to have epidural-like spread pattern.

In this case, the contrast spread pattern after initial injection was clearly non-epidural in the CLO view since it lay posterior to the VILL, and also non-epidural (posterior extension) in the lateral view. In the AP view, the superficial non-epidural pattern appears like epidural spread as shown in Fig. 1. While superficial non-epidural spread in case of false loss of resistance can be determined to be non-epidural by the fact that the spread will be posterior to the VILL and the spinolaminar line in CLO and lateral views, such depth landmark is not available for the AP view. Hence the determination of epidural needle placement in AP view is entirely dependent upon contrast pattern recognition, and as this case report illustrates, this may lead to ambiguity. Although the lateral and CLO views should be equal in their ability to distinguish superficial non-epidural spread, the radiological landmark for the posterior boundary of the epidural space, the VILL, is crisper and as this case report illustrates, the CLO view may provide the highest level of confidence in distinguishing epidural from superficial non-epidural spread. This case report illustrates the importance of obtaining a depth view prior to making the determination of epidural versus non-epidural spread and in case of ambiguity, if the lateral view is primarily employed, a CLO view may provide further clarity. Future studies are essential to validate the utility of AP, Lateral and CLO views in the accurate detection of epidural contrast spread.

The epidurogram in the AP view can demonstrate contrast spread patterns that may result in a false positive, hence this view cannot be relied upon to accurately ensure that the needle placement is in the epidural space. The CLO and lateral views are necessary to confirm true epidural placement. However, the true sensitivity and specificity of these purported depth views with contrast confirmation for epidural needle placement are yet to be determined.

## Funding

None.

## Declaration of competing interest

The authors declare that they have no known conflicts of financial interests or personal relationships to reveal that could have appeared to influence the work reported in this paper.
